# Variety Identification of Corn Seeds Based on Hyperspectral Imaging and Convolutional Neural Network

**DOI:** 10.3390/foods14173052

**Published:** 2025-08-29

**Authors:** Linzhe Zhang, Chengzhong Liu, Junying Han, Yawen Yang

**Affiliations:** College of Information Science and Technology, Gansu Agricultural University, Lanzhou 730070, China; zhanglz@st.gsau.edu.cn (L.Z.); hanjy@gsau.edu.cn (J.H.); 1073323020390@st.gsau.edu.cn (Y.Y.)

**Keywords:** hyperspectral imaging technology, corn seeds, classification, convolutional neural network, non-destructive

## Abstract

Corn as a key food crop, has a wide range of varieties with similar appearances, making manual classification challenging. Thus, fast and non-destructive seed variety identification is crucial for improving yield and quality. Hyperspectral imaging is commonly used for non-destructive seed classification. For the advancement of smart agriculture and precision breeding, in this study, 30 corn varieties from Northwest China were analyzed using hyperspectral images (870–1709 nm) to extract spectral reflectance from the embryonic region. Traditional methods often involve selecting specific bands, which can lead to information loss and limited variety selection. In this study, information loss was reduced and manual intervention was minimized by using full-band spectral data. And preprocessing is performed using first-order derivatives to reduce the interference of noise and irrelevant information. Classification experiments were conducted using KNN, ELM, RF, 1DCNN, and an improved 1DCNN-LSTM-ATTENTION-ECA (CLA-CA) model. The CLA-CA model achieved the highest classification accuracy of 95.38%, significantly outperforming traditional machine learning and 1DCNN models. It is demonstrated that the innovative module combination method proposed in this study is able to successfully classify varieties of corn seeds, which provides a new option for the rapid and non-destructive identification of a variety of corn seeds.

## 1. Introduction

Corn is one of the world’s three major food crops, widely grown around the world, with strong adaptability, high yield and so on [1], it occupies a strategic position in guaranteeing food security and economic development. As one of the most important corn seed production bases, Northwest China is characterized by a wide geographical area, large temperature difference, and few pests and diseases [2]. A wide range of corn varieties are grown in this region, each with different advantages. Therefore, ensuring that seeds suitable for specific regional climate and soil conditions are matched to growers is the key to improving yield and quality. The current variety identification methods based on phenotypic characters are difficult to meet the precise needs for variety purity in the modern seed industry [3]. To address this gap, we propose establishing a non-destructive spectral identification system. This approach is essential for achieving precise germplasm characterization and advancing intelligent agriculture practices.

Traditional corn seed variety identification methods mainly include artificial detection and biological detection methods: artificial detection through the size, shape, color, and other aspects of the corn seed to distinguish between different varieties; biological methods mainly through DNA identification to determine its [4]. Both methods exhibit critical limitations, including insufficient discriminative power for similar seed phenotypes, high subjectivity leading to misclassification, elevated operational costs, and seed damage during testing. Consequently, the breeding industry urgently requires a non-destructive, rapid, and high-accuracy approach for maize variety identification. In recent years, hyperspectral technology is commonly used for variety and quality testing of various crops. The quality of black tea depends largely on its fermentation process. Huang et al. collected hyperspectral data of Yinghong No. 9 black tea during five fermentation time intervals within 0–5 h. The detection model PSO-CNN-LSTM showed the best classification performance with an accuracy of 96.78% on the test set [5]. Huang et al. performed spatially resolved (SR) spectral analysis on 1500 apples from the same orchard. Partial Least Squares Discriminant Analysis (PLSDA) models for single SR spectra and spectral combinations were developed to compare their performance for variety detection. Spectral combinations provided better accuracy in variety detection, with the best overall classification of 99.4% for the spectral ranges in the near-infrared and whole region [6]. Hyperspectral imaging enables non-destructive, rapid maize seed identification essential for modern agriculture. Distinct phenotypic and biochemical variations between varieties manifest as unique spectral signatures, allowing precise classification through spectral curve differentiation.

Traditional machine learning methods are used to deal with complex problems in various domains and have been widely used for classification tasks in hyperspectral imaging techniques, which has become a popular method for non-destructive testing [7]. Zhang et al. manually screened five corn seed varieties with normal quality and flawless appearance, preprocessed the full-band spectra, screened the characteristic wavelengths, and built a model. In the test set, MSC-(CARS-SPA)-GA-SVM has the best performance with an accuracy of 93.00% [8]. Ji et al. used a selective potato classification method. Data dimensionality was reduced using linear discriminant analysis (LDA) and support vector machine (SVM) models were built to classify these types with tested accuracies of up to 90% [9]. Jin et al. obtained hyperspectral images of wheat particles by fusing the 128 bands acquired. Finally, CSKNN is proposed for training and testing of wheat varieties. CSKNN has high classification accuracies of 98.09% and 97.45% [10]. However, the performance of traditional machine learning models on complex data is not satisfactory, and it is difficult to meet the multi-species, multi-band data classification.

In recent years, more deep learning algorithms have been used for the analysis of hyperspectral imaging data in addition to the commonly used traditional machine algorithms [11]. Zhang et al. in order to detect perfect wheat seeds and six imperfect seeds, a method was proposed to recognize imperfect wheat seeds quickly and non-destructively using hyperspectral images. It is shown that the recognition efficiency of MobileNet V2 network model is as high as 97.71% [12]. Polder et al. took hyperspectral images in the field at 5 mm line intervals. A fully convolutional neural network was applied to the hyperspectral images and trained on two experimental rows in the field. The trained network was validated on two additional rows of different potato varieties. For three of the four row/date combinations, precision and recall exceeded 0.78 and 0.88, respectively, compared to traditional disease assessment [13]. Zhang et al. proposed a self-supervised learning method including pre-training and fine-tuning phases using 20 corn seeds as experimental materials. In the seed classification task, the pre-trained model (OA = 99.10%, Kappa = 0.9905) performs significantly better than the corresponding non-pre-trained model (OA = 94.50%, Kappa = 0.9421) [14]. Wang et al. extracted and preprocessed hyperspectral data of 496 seeds with four vigor classes. Finally, 1DCNN, one-1DLSTM, CNN-LSTM, and FA-CNN-LSTM are used to differentiate the spectral images of the vigor grades of sweet corn seeds. The classification accuracy of the proposed FA-CNN-LSTM model in this study is 97.23% [15]. Huang et al. used hyperspectral imaging to detect mechanical damage in corn seeds and were classified into four categories. Three feature extraction methods and a joint feature extraction method that combines these methods were compared. Then compare the effectiveness of six classification models and propose ResNeSt_E based on ResNeSt_E and an efficient multi-scale attentional modular network, which can achieve 99.0% accuracy [16]. Li et al. first used LDA to downscale hyperspectral corn seed images. Second, fine-grained features are extracted from the images using effective residual blocks. Finally, utilize a classifier to detect and classify hyperspectral corn seed images. The accuracy of ERNet is 98.36% [17], although it has good classification effect, only 10 varieties of corn seeds were available in this study, which is difficult to meet the current situation of multiple types of corn seeds in the market.

In existing studies, the classification of corn varieties usually uses fewer varieties, which is difficult to ensure under the realistic conditions of many corn varieties; there is still a better classification effect. In most studies, the operation of selecting the characteristic wavelengths is performed first [18], which can reduce the influence of non-correlated variables and improve the model performance, but it also means that it may lead to the loss of some spectral information. Therefore, this study chose to use 30 different varieties of corn as the research object, which is close to the actual situation. At the same time, the experiment is carried out using the whole waveband to avoid the loss of spectral information, and it can reduce the influence of human operation and simplify the experimental operation. The deep learning model and the attention mechanism in the above study have good performance effects in various aspects, so this study uses 1DCNN as the basic network model, and adds LSTM and attention mechanism for improvement, in order to achieve the purpose of accurate classification.

## 2. Materials and Methods

### 2.1. Experimental Materials

The corn seeds used in this experiment were all provided by the Gansu Provincial Academy of Agricultural Sciences (GSAAS), and 30 varieties of corn seeds widely planted in Northwest China were selected, such as Early A Shengyuan 688 (ZASY688), Zhongshui A Longxin District 518 (ZSALXQ518), Early A Qiangfeng 216 (ZAQF216), and Early B Aerospace 526 (ZBHT526), etc., and were labeled from 1 to 30, respectively. In order to avoid the effect of moisture in the air and keep the seeds dry, the corn seeds were stored in sealed paper bags, and 30 intact and undamaged corn seeds without obvious insect pests were selected for each variety for each sampling for hyperspectral image collection. The corn varieties are shown in Table 1.

### 2.2. Experimental Equipment

**Figure 1:**
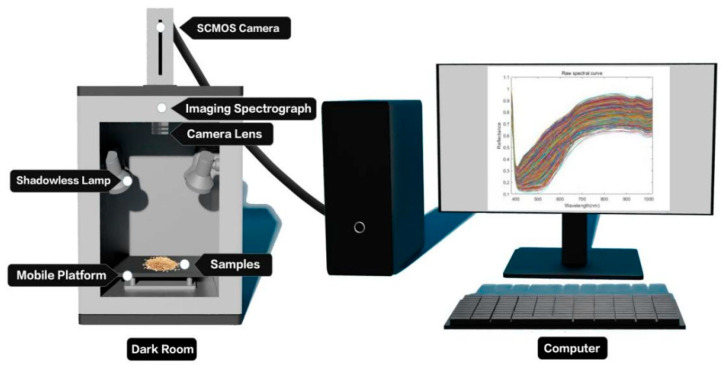
Hyperspectral imaging system. On the left is the Hyperspectral Dark Room, which includes an SCMOS Camera, an Imaging Spectrograph, a Camera Lens, a Shadowless Lamp, and a Mobile Platform. On the right is the Computer. The Dark Room is connected to the Computer, which is used for image acquisition.

The GaiaField portable hyperspectral system (Sichuan Dualix Spectral Imaging Technology Co., Ltd.) is shown in Figure 1, which includes a GaiaField-V10E hyperspectral camera, a 2048 × 2048 pixel imaging lens, an HSI-CT-150 × 150 standard whiteboard (PTFE), an HSIA-DB indoor imaging dark box, four sets of shadowless lamp light sources, an HSIA- TP-L-A tripod rocker arm set, and hyperspectral data acquisition software Spec View. The spectral range is 870–1709 nm, the spectral band is 512, the spectral resolution is 2.8 nm, the numerical aperture is F/2.4, the slit size is 30 mm × 14.2 mm, the detector is SCMOS, and the imaging modes are built-in push-scan, autofocus, and the dynamic range is 14 bits. The core components of the hyperspectral equipment include a standardized light source, a spectral camera, an electronically controlled mobile platform, a computer and a control software. The system works by using a push-scan imaging mode that combines a surface array detector with an imaging spectrometer. Driven by the scanning-controlled motorized platform, the slit of the imaging spectrometer and the focal plane of the imaging lens move relative to each other, and the detector collects real-time information relative to the line target, which is finally stitched together into a complete data cube.

### 2.3. Acquisition and Correction of Hyperspectral Images

**Figure 2:**
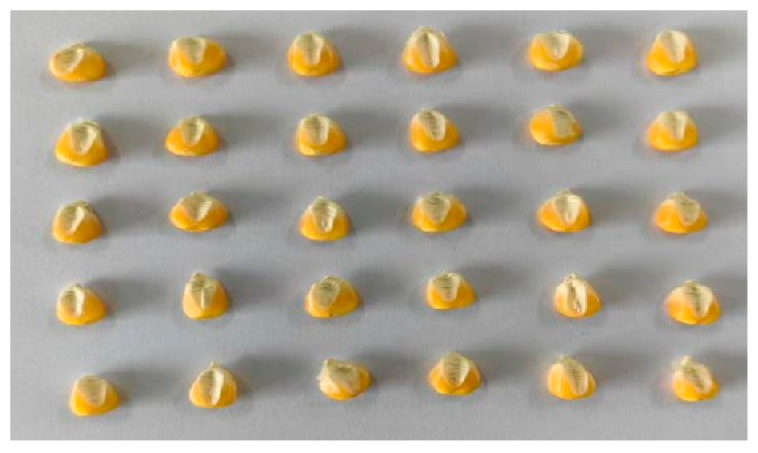
Corn seed placement chart. Arrange in a 6 × 5 pattern.

Before image acquisition, the hyperspectral instrument switch and dark box light source were set. A warm-up time of 30 min was allowed, and then the instrument parameters were configured to set the camera exposure time to 49 ms, gain to 2, frame rate to 18.0018 Hz, and forward speed to 0.00643 cm/s. Corn seed embryos contain a variety of nutrients and play a key role in providing nutrition during the corn germination process; therefore, in this experiment, we collected image information of the embryonic surface of the samples. We chose a total of 30 corn seeds; each hyperspectral image was collected a total of three times, each time 30 seeds were randomly selected from the corresponding varieties and embryo-surface-facing were placed in a dark box on a mobile platform for collection. After each variety was collected once, the sample under test was re-poured into the sample bag and shaken manually to homogenize it. Corn seeds were arranged in a 5 × 6 array for hyperspectral imaging, arranged as shown in Figure 2. This procedure was repeated three times per variety, totaling 90 seeds per variety. Ultimately, 2700 spectral curves were obtained. In order to improve the stability and reliability of the images, after the acquisition was completed, the original hyperspectral images were black- and white-corrected to eliminate the effects caused by dark current noise [19]. The formula for black and white correction is as follows:

(1) $$I_{raw}=\frac{I_{raw}-I_{dark}}{I_{white}-I_{dark}}$$

where *I_raw_* is the original image, *I_white_* is the white reference image, *I_dark_* is the dark reference image, and *I_c_* is the calibration image.

To extract spectral information from the corrected hyperspectral images, spectral features were extracted using ENVI5.3 software with the embryo of each corn seed in a single image as the region of interest.

### 2.4. Spectral Pre-Processing

The spectral information of a single band or a limited number of bands may not be comprehensive enough, which may easily lead to the misjudgment or omission of the target, while the full-band data provides richer information. To be able to reduce this uncertainty, this study no longer performs feature wavelength extraction and uses all 512 bands for classification experiments as a way to obtain more comprehensive spectral information. This operation is performed directly by the machine to learn the data, which reduces the possible influence of human operation and improves the reliability of classification. And compared with the traditional method of extracting feature wavelengths, it reduces the manual workload, simplifies the operation steps, and only needs to be put into the model once. Although it increases the complexity and computation of data processing, with the continuous improvement of computer power, machine learning, and deep learning algorithms, the model is more advanced, and the application of this method will be more extensive.

In the process of using hyperspectral instruments to collect images, it is inevitable to be interfered by instrumental noise, environmental noise, etc. In order to reduce the interference of extraneous information, improve the quality of the obtained data, and make the acquired spectral data better reflect the sample information, and ensure that the established model obtains more accurate prediction results, it is necessary to carry out the preprocessing of spectral data. In this study, the first-order derivative (1st Der), second-order derivative (2nd Der) [20], polynomial smoothing (SG) [21], Gaussian filter (GF) [22], median filter (MF) [23], and moving average filter (MA) [24] are used as preprocessing methods to preprocess the raw spectral data. The first-order derivatives can keenly capture the trend of data changes, amplify the information such as the change in curve slope, and highlight the increase or decrease in data at each point, which is conducive to analyzing the dynamic characteristics of the data [25]. To a certain extent, it can weaken the noise interference in the smooth region, make the details of the changes originally covered by noise clearer, improve the quality of data, and facilitate the subsequent accurate analysis and modeling. Converting the original data into derivative form can simplify the data expression by retaining the key change characteristics, reducing data redundancy, and improving processing efficiency.

**Figure 3:**
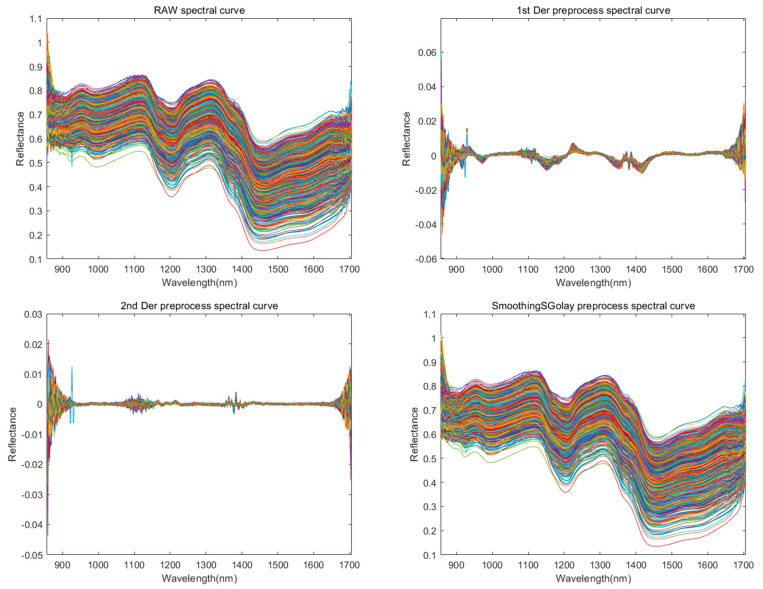

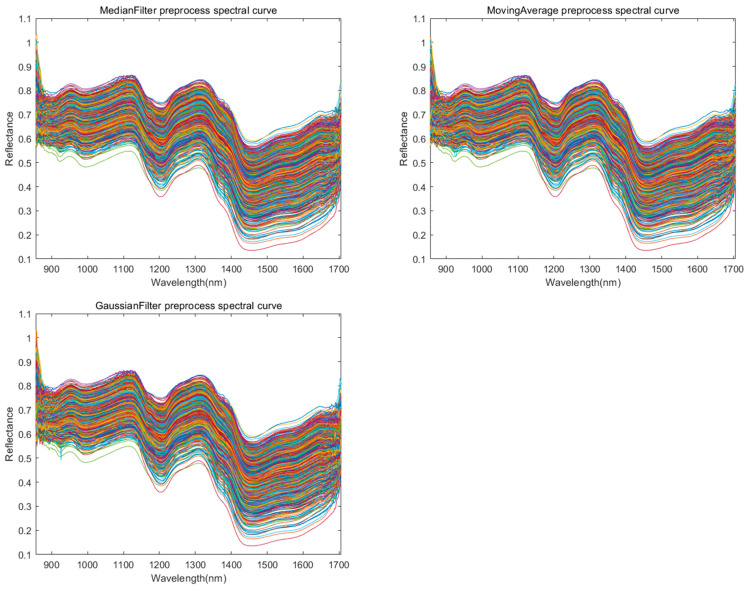
Spectral reflectance curve of corn. Representing the original image (RAW spectral curve), first-order derivative (1st Der preprocess spactral curve), second-order derivative (2nd Der preprocess spectral curve), and spectral images after preprocessing operations such as SG (Smoothing SGolay preprocess spectral curve), MF (Median Filter preprocess spectral curve), MA (Moving Average preprocess spectral curve), GF (Gaussian Filter preprocess spectral curve), etc.

The waveform plots of the original data and the six preprocessing methods are shown in Figure 3.

### 2.5. Division of Training and Test Sets

In this experiment, 2700 samples were divided into training and test sets in the ratio of 4:1, and the training and test sets for each corn seed were 72 and 18, respectively. The training and test sets for the 30 species were 2160 and 540, respectively, in order to analyze and compute the discriminative accuracy of the training and test sets of the model.

### 2.6. Experimental Environment

**Figure 4:**
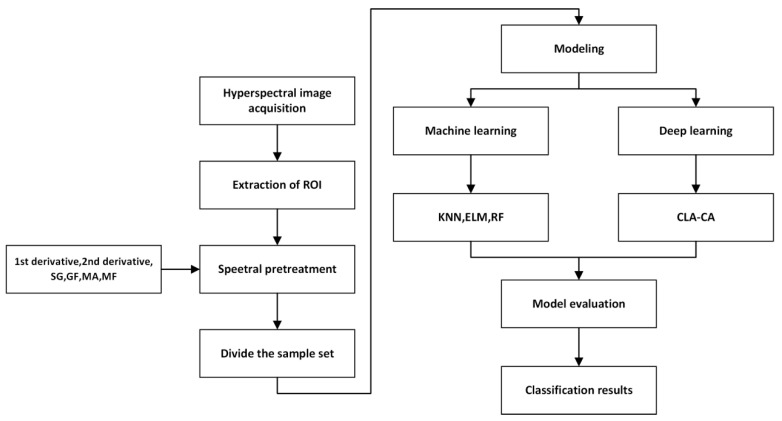
Flow chart of the experiment. This process begins with acquiring hyperspectral images, followed by extracting regions of interest to obtain spectral curves and performing preprocessing. Subsequently, the dataset is partitioned and classified using different models, with the classification results compared.

The machine learning model code was implemented using Matlab R2023a, and the deep learning model code was developed and researched using Python 3.9 programming language, Pytorch environment. The running environment of the model is Windows 11 system, and the hardware information is as follows: CPU: 13th Gen Intel(R) Core(TM) i7-13700 2.10 GHz; GPU: NVIDIA GeForce RTX 4090. The whole experimental process is shown in Figure 4.

### 2.7. Traditional Machine Learning Models

The K-Nearest Neighbors (KNN) algorithm is a commonly used supervised learning algorithm that is widely used for classification and regression tasks [26]. The basic idea is that for a new data point, the KNN algorithm looks for the K nearest neighbors to it in the training set and then predicts the category of the data point based on the categories or values of these K neighbors. The value of K is an important hyperparameter indicating the number of nearest neighbors to be taken into account in the prediction. In general, the smaller the K value, the more sensitive the algorithm may be to noise, and the larger the K value, the smoother the algorithm will be, but it may lose its sensitivity to local structure. Selection of the appropriate K value is usually achieved by cross-validation.

Extreme Learning Machine (ELM) algorithm is a method for training single hidden layer feedforward neural networks [27]. In traditional neural network training, we need to optimize the weights and biases of the network by back propagation algorithm, and this process is usually computationally intensive and time consuming. In ELM, on the other hand, the hidden layer weights and biases of the network are randomly generated without updating, and only the weights of the output layer need to be optimized by least squares. This method can significantly improve the training efficiency and avoid the local optimum problem.

Random Forest (RF) is a commonly used integrated learning algorithm widely used for classification, regression, and other machine learning tasks [28]. It improves the accuracy and stability of the model by constructing multiple decision trees, which solves the problem of overfitting that can easily occur with a single decision tree. Random forest is an integrated learning method, specifically, it belongs to an instance of bagging method. The basic idea is to make a final decision by constructing multiple decision trees and by integrating the results of these trees.

### 2.8. Deep Learning Models

One-dimensional convolutional neural network (1DCNN) is a kind of deep learning model, which is widely used in the fields of image recognition, target detection, and natural language processing [29]. 1DCNN uses a convolutional layer to extract features from sequential data. A convolutional layer performs a convolutional operation on the input data by sliding a fixed-size window, extracting features within the window, and then mapping those features to the next layer. One-dimensional convolutional neural networks can extract localized features in text and classify them using a fully connected layer. One-dimensional convolutional neural networks can effectively extract local features in the input sequence, which is very important for processing sequence data. 1DCNN utilizes the characteristics of local awareness and weight sharing, which reduces the number of parameters of the model, reduces the computational complexity, and improves the computational efficiency and accuracy. It can also be used in combination with other deep learning models to handle more complex tasks. In this study, the data extracted from hyperspectral images are sequence data, so we choose to use 1DCNN as the base model for improvement.

The sequence data used in this study contains 512 bands, which are long sequence data, so long short-term memory network is added to capture the long-term dependencies to improve the experiment. Long short-term memory network (LSTM) is a special kind of recurrent neural network (RNN). The RNN unfolds and consists of multiple identical units connected consecutively. However, the actual structure of the RNN is a self-constant loop structure, i.e., with the increasing input data, the above self-cycling structure passes the previous state to the current input, together with the new input data for the current round of training and learning, until the input or training is finished, and the final output obtained is the final prediction result. The LSTM mainly comprises input gate, forgetting gate, and output gate; through the introduction of the “gate” mechanism, it solves the problem of gradient disappearance or gradient explosion, which is easy to occur in traditional RNN when dealing with long sequences so that the LSTM is able to more effectively capture long-term dependencies [30].

Due to the large number of bands and the fact that the selection of characteristic bands is no longer performed in this study, the attention mechanism is introduced so that the model focuses more on the portion of the bands that it considers important. Attention mechanism is a technique in deep learning, especially widely used in the field of natural language processing and computer vision. Its core idea is to mimic the human attention mechanism, i.e., humans will focus their attention on certain key parts when processing information and ignore other less important information. In natural language processing tasks, the input data is usually in the form of text, and we need to convert this text into a numerical form that the model can handle. This process is called embedding. The embedding layer maps each word to a vector in a high-dimensional space; these vectors are called word vectors. Word vectors capture semantic information about words and can be processed by neural networks. In machine learning models, this can help the model to better capture the key information in the input data.

In order to meet the actual classification needs, the channel attention module is added in this study to improve the classification effect. ECA attention module, which is a channel attention module; is often applied with visual modeling. Based on the SE module, the effects of channel dimensionality reduction and cross channel interaction on channel attention are re-examined, and a more efficient ECA channel attention is proposed. The ECA module firstly determines the size of the kernel adaptively after aggregating convolutional features using the GAP layer, then performs a one-dimensional convolution, and finally utilizes the Sigmoid function to learn channel attention and ultimately generates the attention graph. Compared with the SE module, the ECA module more effectively avoids the problem of dimensionality reduction and thus is able to capture the interaction information between channels more effectively.

**Figure 5:**
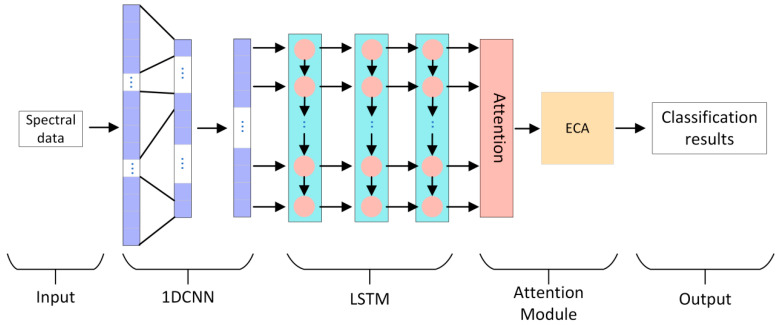
CLA-CA structure. Including the Input layer, 1DCNN, LSTM, Attention mechanism, ECA module, and Output layer.

**Figure 6:**
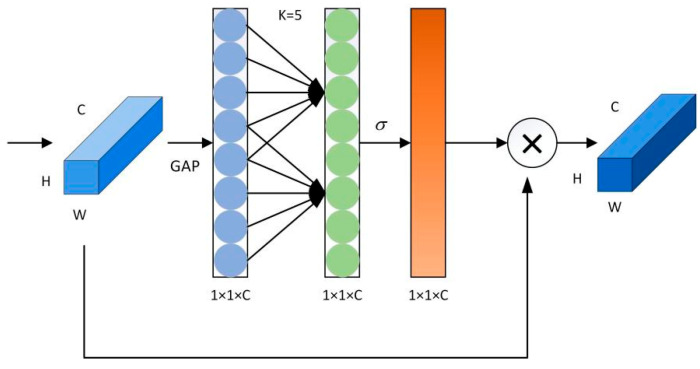
ECA structure. Based on the aggregated features obtained via Global Average Pooling (GAP), ECA generates channel weights by performing fast one-dimensional convolutions of size k, where k is adaptively determined through mapping the channel dimension C.

In this paper, a new deep learning network model 1DCNN-LSTM-ATTENTION-ECA (CLA-CA) is proposed. The network model includes 1DCNN, LSTM, an ATTENTION mechanism and an ECA module, where 1DCNN is a one-dimensional convolutional neural network (1DCNN), which specializes in acquiring local features of data, including an input layer, a convolutional layer, a pooling layer, and an output layer. In this study, all 512 bands are used as input data, and kernel_size, stride, and padding are set to 3, 1, 1, respectively. The initial learning rate is set to 0.001, and batch_size is set to 16, with a total of 200 iterations. While LSTM is good at capturing long-term dependencies, and 1DCNN both can complement each other’s strengths and are more suitable for the sequence data in this study, the number of LSTM layers is 3. The ATTENTION mechanism can automatically pay attention to the important parts of the data; ATTENTION number of heads is 8. And the addition of the ECA module enhances the ability of the network to pay attention to the key features, which avoids the loss of performance caused by the possible dimensionality reduction operation in the channel attention mechanism, and achieves channel weighting through an adaptive cross channel interaction strategy to realize the generation of channel weights and improve the accuracy of the classification [31]. The network structure diagrams of CLA-CA and ECA are shown in Figure 5 and Figure 6, respectively:

RELU Function: In this study, the RELU function is used as the activation function. This function is widely used in deep learning models as a commonly used linear activation function, it is much faster to compute RELU compared to traditional neural network activation functions, such as the sigmoid function and the tanh function, so it can improve the training efficiency [32].

ADAM Optimizer: the ADAM optimizer is used in this study for network optimization. This optimizer combines Momentum and Adaptive Learning Rate methods (e.g., AdaGrad, RMSProp) [33], which can adjust the learning rate according to the historical gradient and squared gradient of each parameter.

### 2.9. Model Evaluation Indicators

The classification of corn varieties in this study is a multi-category classification task with the same number of samples in each category, and the accuracy rate is applicable to a data set with a balanced distribution of categories and is able to indicate the proportion of the number of correctly classified samples to the total number of samples. Precision rate indicates the ratio of the number of true examples to the number of all samples predicted to be positive examples and evaluates the ability of the classifier to recognize positive examples. Recall rate indicates the proportion of the number of true examples to the number of samples of all actual positive examples and evaluates the classifier’s ability to classify positive examples. Therefore, accuracy, precision, and recall are used as the evaluation metrics of the model in this study [34].

The three formulas are shown in Equations (2)–(4). Where TP is the true example, FN is the false negative example, FP is the false positive example, and TN is the true negative example.

(2) $$Accuracy=\frac{TP+TN}{TP+TN+FP+FN}$$

(3) $$\mathrm{Pr}ecision=\frac{TP}{TP+FP}$$

(4) Recall=TPTP+FN

In this study, cross entropy is used as the loss function of the model, which focuses on the degree of discrepancy between the predicted and true values of the model, and it is fast to learn when the model is less effective, and it is suitable for multiclassification models [35]. The loss function formula is shown in Equation (5).

(5) $$Loss=-\frac{1}{N}\sum_{i}\sum_{c=1}^{M}y_{ic}\log(p_{ic})$$

## 3. Results

Since the traditional approach in hyperspectral classification experiments uses different data preprocessing methods and machine learning model permutations to find the optimal method, it is important to compare the effectiveness of traditional machine learning models such as KNN, ELM, RF, and the deep learning model 1DCNN, as well as the improved CLA-CA based on 1DCNN in the classification task of the present study so as to select a more suitable classification model. In the KNN classification model, different preprocessing methods are used to preprocess the data, and the K values are taken as 1, 3, 5, 7, 9, 11, 13, and 15. The normalization of the data features are used as the mean, maximum and minimum, logarithmic, etc., the distance metrics are used as the Euclidean, Manhattan, and Spearman, and correlation distances as the evaluation indexes for the classification effect. The results show that the data preprocessed using SG had the best results when using the maximum–minimum normalization process, K value of 5, and using the correlation distance as the distance metric; however, it was only 48.33%, and the results show that using the KNN model does not classify the data in this study. The best results of other preprocessing are shown in Table 2.

Since its maximum classification accuracy is still not more than 50%, it can be concluded that the use of KNN model cannot classify corn seeds in this study.

Secondly, the classification is performed using the ELM model. In the ELM model, the number of hidden layer nodes are set as 100, 150, and 200, respectively. It is found that the highest classification accuracy is achieved when the number of hidden layer nodes is taken as 150, and the highest accuracy that can be achieved is 68.07% when using SG preprocessing, and thus it is set as 150. The accuracy of this model under different preprocessing methods is shown in Table 3:

Then the classification experiment was conducted using RF model. In the RF model, the number of decision tree was set to 50, 100, and 150, and the minimum number of leaves was set to 1, 2, and 4, respectively. It was found that the classification effect was the best when the number of decision trees was set to 100, and the minimum number of leaves was set to 1. Therefore, the experiment was carried out using these two parameters, and its accuracy could be the highest when preprocessed using 1st Der to reach 69.44%. The accuracy of this model under different preprocessing methods is shown in Table 4.

Next, this study used the 1DCNN model for classification as the classification accuracy was much higher than the classification results of the other three machine learning models. Since the preprocessing methods that can achieve the highest classification accuracy are not the same in different machine learning models, it is considered that the preprocessing methods applicable in different models are also different. Therefore, we first study the effect of different preprocessing methods on the experiments and compare the original data and the data preprocessed by five different methods in one layer of convolutional 1DCNN, respectively. It was found that the accuracy of the test set preprocessed by the first-order derivative (1st Der) is the highest, reaching 90.27%, which is higher than that of the original data (79.80%), an improvement of 10.47%, and both the precision and recall are higher than those of the other preprocessing methods; model runtime outperforms most other preprocessing methods, which proves that the first-order derivative for preprocessing can improve the accuracy, precision, and recall of classification. Therefore, this study chooses the first-order derivative to preprocess the original data. The preprocessing results are shown in Table 5.

**Figure 7:**
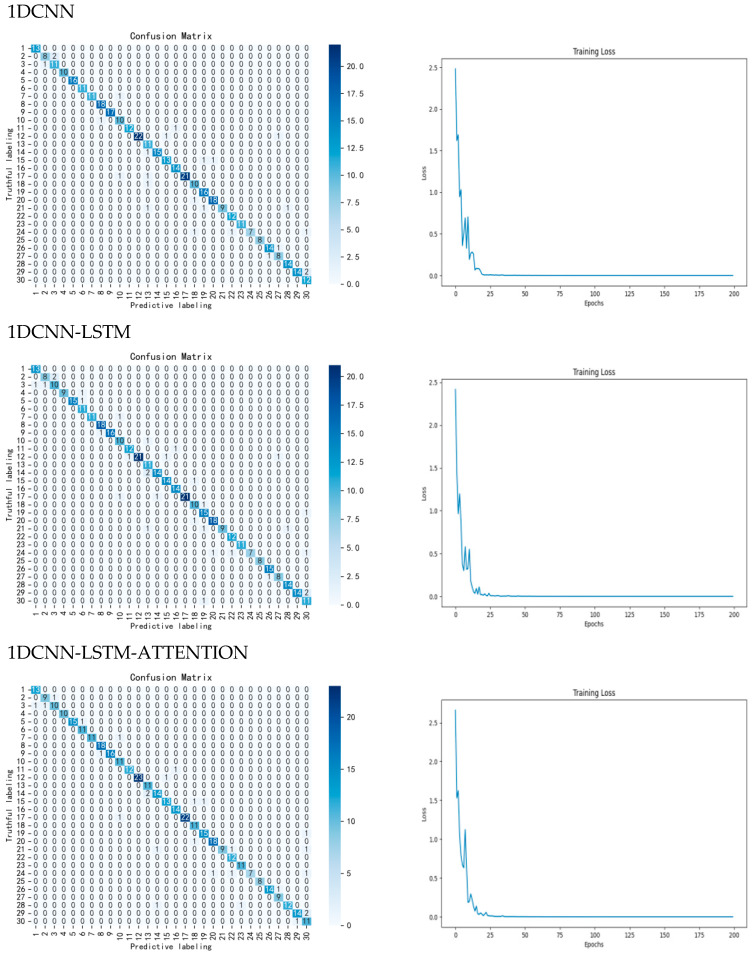

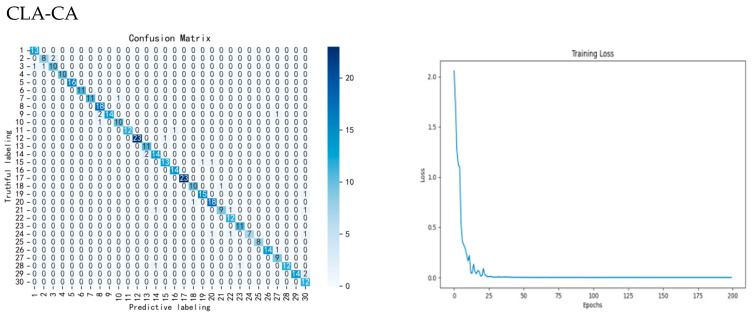
Confusion matrix and loss function. The confusion matrices and loss functions for 1DCNN, 1DCNN-LSTM, 1DCNN-LSTM-ATTENTION, and CLA-CA are displayed separately. The left side shows the confusion matrix, while the right side displays the loss function, collectively presenting the classification performance of each model.

Secondly, ablation experiments are conducted on different parts of the model to test the effect of adding parts such as LSTM, ATTENTION, and ECA modules to the 1DCNN on the experimental results, respectively. Firstly, by adding LSTM to the CNN network model after first-order derivative preprocessing, the accuracy of the test set is improved from 90.27% to 92.94%, which is an improvement of 2.67%, and the precision and recall are improved by 1.64% and 2.30%, respectively. Then, when ATTENTION is added into the network model, the accuracy is improved to 93.43%, which is an improvement of 0.49%, and the precision and recall are improved by 1.36% and 0.40%, respectively, proving that LSTM and ATTENTION can work together in CNN network, and both of them can improve the classification accuracy of the overall network at the same time. Therefore, this study chooses CNN-LSTM-ATTENTION as the base network model and adds the ECA module on top of it to construct the model CLA-CA used in this study. After adding this module, the overall model accuracy is increased to 95.38%, which is an overall improvement of 1.95%, and the precision and recall are increased by 0.14% and 0.84%, respectively, the model runtime did not increase much compared to the original model, which proves that the ECA module is able to focus on different channel features, thus improving the accuracy of model classification. The results and loss function of the confusion matrix are shown in Figure 7. The ablation results are shown in Table 6.

After the experiments were completed, the best results of each model were compared and discussed, and it was determined that the CLA-CA model worked best on the classification task in this study and outperformed all the compared models. A comparison of the best results for each model is shown in Table 7.

Compared with existing corn variety classification studies, the number of corn varieties in this study is closer to the actual situation. While increasing the number of corn varieties, this study maintains a high accuracy rate. Compared with existing studies that also use the full wavelength range, this study improves the accuracy of variety classification. Additionally, the model used in this study has a shorter runtime. Compared with the traditional methods used in existing studies, this study eliminates the time required for feature wavelength screening. In summary, this study has significant advantages in terms of variety number, accuracy, and runtime.

## 4. Discussion

This study takes 30 varieties of corn seeds as the research object, discusses the classification effect of different models and different preprocessing methods.

In this study, first-order derivative (1st Der), second-order derivative (2nd Der), polynomial smoothing (SG), Gaussian filter (GF), median filter (MF), and moving average filter (MA) were used as preprocessing methods to preprocess the raw spectral data. It was found that the first-order derivative preprocessed data had obvious advantages in terms of accuracy, precision, recall, and runtime, so this study finally chose to use the first-order derivative for preprocessing.

The effect of each part of the network model improvement on the accuracy of the model and the results show that the classification effect after using 1DCNN and preprocessing the data is much higher than that of the machine learning models such as KNN, ELM, RF, etc., reaching above 90%. This proves that the classification model is feasible in this study. On the other hand, the machine learning model has a large difference in classification accuracy, making it difficult to achieve accurate classification and realize the demand of seed production. After each improvement to the 1DCNN model, the classification effect can be further improved, proving that each improvement to the network is feasible and can bring about an improvement in the classification effect in the study, so it is determined that the use of the improved CLA-CA model can effectively classify 30 varieties of corn seeds in the study, and the classification accuracy can reach 95.38%, with a precision rate and a recall rate of 94.26% and 94.16%, respectively, and thus realizing the precise classification of corn seeds.

In contrast to existing studies on corn variety classification, the number of varieties examined in this study more closely approximates real-world conditions. Critically, while increasing the number of varieties, the approach maintains a high classification accuracy [17]. Compared with existing studies that also use the full wavelength range, this study improves the accuracy of variety classification [8]. Furthermore, the model employed demonstrates reduced computational time. Unlike traditional methods used in prior research, it eliminates the need for feature wavelength screening, thereby saving significant processing time [16]. Consequently, this study demonstrates significant improvements in terms of the number of varieties classified, classification accuracy, and computational efficiency. Therefore, the methods used in this study can be applied to the intelligent breeding industry to help establish a non-destructive identification system, improve identification efficiency and accuracy, thereby achieving precise management of germplasm resources and promoting the development of smart agriculture.

This study has several limitations: (1) current equipment constraints limit experimentation with broader spectral bands; (2) exploration of additional preprocessing methods is needed; (3) measurement of physicochemical indicators for comprehensive seed quality validation remains outstanding. We will carry out related work in the future.

## 5. Conclusions

(1) This study presents an efficient and automated approach for maize seed variety identification based on hyperspectral imaging. By analyzing 30 widely cultivated varieties from Northwest China—a sample size that better reflects real-world diversity than previous studies—we avoided conventional feature wavelength selection, thereby minimizing information loss and subjective bias through direct processing of full 512-band spectral data.
(2) In order to solve the problem of traditional machine learning that has difficulty coping with complex data, especially the many varieties of corn seeds—this study uses the full-band data, which increases the complexity of the data—this study uses the 1DCNN in the deep learning model as the basic model and improves it, so as to satisfy the classification needs of this study. The results show that the classification accuracy of the 1DCNN model with first-order derivative preprocessing but without improvement can reach 90.27%, which can meet the requirements of the classification task. Further innovations integrating LSTM, ATTENTION, and ECA modules yielded the CLA-CA model, achieving a significantly higher accuracy of 95.38%. The precision rate and recall rate are 94.26% and 94.16%, respectively. These findings underscore the potential of deep learning-driven hyperspectral analysis as an accurate and non-destructive tool for seed purity inspection, contributing significantly to precision breeding and quality control in modern agriculture.
(3) This study utilized the spectral range of 870–1709 nm. Future work will employ cameras with broader spectral ranges to collect data and compare the effect of different spectral ranges on maize seed variety classification accuracy, thereby enhancing experimental scalability. To minimize spectral information loss, the full spectrum was used. Subsequent studies will investigate a combined approach of full spectrum and feature wavelength selection to balance efficiency with accuracy. And the collected data can also be used for subsequent seed quality testing to guarantee seed quality.

## Figures and Tables

**Table 1:** Corn varieties.

No.	Name	No.	Name	No.	Name
1	ZASY688	11	GMBSB1259	21	ZHAXY99
2	ZSASQ66	12	ZBQF218	22	ZHBP220
3	ZSALXQ518	13	QAGNY599	23	ZSBJYY1207
4	ZQBSY438	14	JBL3712	24	ZSAZT2208
5	ZSPY128	15	JAM1910	25	QBFLQC2
6	ZAQF216	16	ZSDTY71110	26	JAC1219
7	ZBHT526	17	ZAQKY918	27	QBFY166
8	ZBJL6	18	GADF1908	28	ZAZD1568
9	GBJH25	19	JAGNY168	29	ZSBZT2209
10	ZJNY516	20	ZBNF7019	30	QBFLQC5

**Table 2:** KNN classification results with different preprocessing.

Pre-Processing Methods	Normalization Method	K-Value	Distance Measurement Method	Classification Results
raw data	average value	7	Spielman Distance	44.82%
1st Der	average value	13	Manhattan Distance	47.96%
2nd Der	average value	15	Relevant distance	24.17%
SG	minimum and maximum values	5	Relevant distance	48.33%
MF	minimum and maximum values	7	Spielman Distance	47.22%
MA	minimum and maximum values	7	Relevant distance	47.03%
GF	average value	7	Spielman Distance	47.78%

**Table 3:** ELM classification results with different preprocessing.

Pre-Processing Methods	Training Set Accuracy	Test Set Accuracy
raw data	88.75%	55.74%
1st Der	80.59%	56.61%
2nd Der	70.52%	38.86%
SG	83.78%	68.07%
MF	79.19%	64.58%
MA	82.74%	66.69%
GF	78.22%	63.37%

**Table 4:** Results of RF classification with different preprocessing.

Pre-Processing Methods	Training Set Accuracy	Test Set Accuracy
raw data	89.95%	35.18%
1st Der	98.74%	69.44%
2nd Der	93.87%	52.22%
SG	88.76%	37.75%
MF	88.53%	35.52%
MA	88.68%	34.01%
GF	89.94%	38.63%

**Table 5:** 1DCNN classification results with different preprocessing.

Pre-Processing Methods	Accuracy	Precision	Recall	Loss	Time
Raw data	79.80%	80.57%	79.32%	1.01e-07	110.43 ms
1st Der	90.27%	91.12%	90.62%	2.84e-07	108.79 ms
2nd Der	85.64%	86.01%	85.84%	1.83e-07	138.92 ms
SG	85.40%	86.26%	86.63%	2.29e-07	117.65 ms
MF	83.45%	84.41%	83.17%	3.03e-07	125.43 ms
MA	80.53%	81.18%	80.27%	1.28e-07	148.92 ms
GF	80.05%	81.38%	79.96%	1.74e-07	101.13 ms

**Table 6:** Results of ablation experiments.

Model	Accuracy	Precision	Recall	Loss	Time
1DCNN	90.27%	91.12%	90.62%	3.03e-07	108.79 ms
1DCNN-LSTM	92.94%	92.76%	92.92%	5.50e-08	124.66 ms
1DCNN-LSTM-ATTENTION	93.43%	94.12%	93.32%	6.42e-08	121.74 ms
CLA-CA	95.38%	94.26%	94.16%	4.58e-08	127.72 ms

**Table 7:** Comparison of the best results of the models.

Model	Accuracy
KNN	48.33%
ELM	68.07%
RF	69.44%
1DCNN	90.27%
CLA-CA	95.38%

## Data Availability

Data available on request due to restrictions. The data presented in this study are available on request from the corresponding author due to the data being collected and obtained by individuals.
